# The L76V mutation in HIV-1 protease is potentially associated with hypersusceptibility to protease inhibitors Atazanavir and Saquinavir: is there a clinical advantage?

**DOI:** 10.1186/1742-6405-8-7

**Published:** 2011-02-13

**Authors:** Frank Wiesmann, Jan Vachta, Robert Ehret, Hauke Walter, Rolf Kaiser, Martin Stürmer, André Tappe, Martin Däumer, Thomas Berg, Gudrun Naeth, Patrick Braun, Heribert Knechten

**Affiliations:** 1 PZB Aachen, HIV&Hepatitis Research Group, Blondelstr., 52062 Aachen, Germany; 2 University of Erlangen, Institute for Clinical and Molecular Virology, Schloßgarten, D-91504 Erlangen, Germany; 3 University of Cologne, Institute for Virology, Fürst Pückler Str. 56, D-50925 Cologne, Germany; 4 University of Frankfurt, Institute for Virology, Paul-Ehrlich-Str. 40, D-60596 Frankfurt, Germany; 5 Roche Pharma, Clinical Project Management, Emil-Barell-Str. 1, D-79639 Grenzach-Wyhlen, Germany; 6 Laboratories Thiele, Institute for Immunology and Genetics, Hellmut-Hartert-Str. 1, D-67655 Kaiserslautern, Germany; 7 Medical Laboratories Berg, HIV Research, Seestr. 13, D-13353 Berlin, Germany

## Abstract

**Background:**

Although being considered as a rarely observed HIV-1 protease mutation in clinical isolates, the L76V-prevalence increased 1998-2008 in some European countries most likely due to the approval of Lopinavir, Amprenavir and Darunavir which can select L76V. Beside an enhancement of resistance, L76V is also discussed to confer hypersusceptibility to the drugs Atazanavir and Saquinavir which might enable new treatment strategies by trying to take advantage of particular mutations.

**Results:**

Based on a cohort of 47 L76V-positive patients, we examined if there might exist a clinical advantage for L76V-positive patients concerning long-term success of PI-containing regimens in patients with limited therapy options.

Genotypic- and phenotypic HIV-resistance tests from 47 mostly multi-resistant, L76V-positive patients throughout Germany were accomplished retrospectively 1999-2009. Five genotype-based drug-susceptibility predictions received from online interpretation-tools for Atazanavir, Saquinavir, Amprenavir and Lopinavir, were compared to phenotype-based predictions that were determined by using a recombinant virus assay along with a Virtual Phenotype™(Virco). The clinical outcome of the L76V-adapted follow-up therapy was determined by monitoring viral load for 96 weeks.

**Conclusions:**

In this analysis, the mostly used interpretation systems overestimated the L76V-mutation concerning Atazanavir- and SQV resistance. In fact, a clear benefit in drug susceptibility for these drugs was observed in phenotype analysis after establishment of L76V. More importantly, long-term therapy success was significantly higher in patients receiving Atazanavir and/or Saquinavir plus one L76V-selecting drug compared to patients without L76V-selecting agents (p = 0.002).

In case of L76V-occurrence ATV and/or SQV may represent encouraging options for patients in deep salvage situations.

## Background

The reduced susceptibility to certain antiretrovirals is often accompanied with a gradual loss of viral fitness, indicating that mutations with high fitness costs are less able to persist in the absence of drug pressure [1]. There have been recent reports about HIV strains with increased susceptibility to particular drugs when certain mutation patterns had developed under antiretroviral treatment [2-5]. This biological attribute enables new putative strategies for future treatment of HIV-infected patients with abundant resistance mutations by trying to take advantage of particular mutations [6].

As example, M184V/I, the most prevalent NRTI-mutations selected under 3TC or FTC in the reverse transcriptase, do for instance revert partially the effect of thymidine-analogue mutation- (TAM) on resistance [7]. K65R and L74V are further mutations which can confer hypersusceptibility or resensitization to AZT [8]. Beside these specific mutations in the reverse transcriptase, there are also reports about resensitizing mutations affecting the protease gene [9,10].

## Objectives

This article reports about possible clinical advantages of a valine substitution, instead of leucine, at position 76 in the HIV-1 protease. This mutation generally disappears quickly in replicating viruses in absence of selection pressure mediated by LPV, APV or DRV treatment. Thus, for deep salvage therapy situations in patients with strongly limited therapy options, it might be of advantage to maintain these drugs in treatment regimens to preserve L76V in the current replicating virus in combination with a "resensitized" drug ATV or SQV.

## Results

### Patients with protease gene mutation L76V show increased susceptibility for Atazanavir and Saquinavir

**Figure 1:**
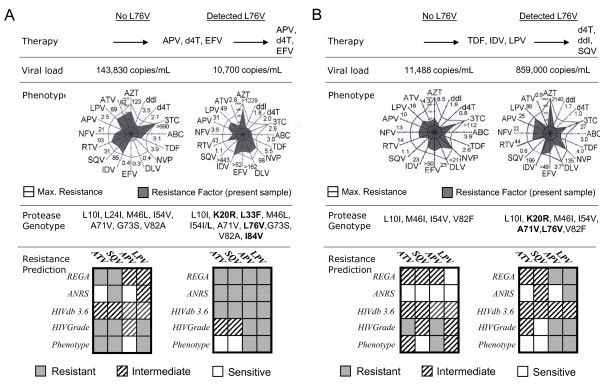
**Resensitizing effects of the L76V mutation are visible in phenotype results: Phenotypic resistance analysis before and after manifestation of L76V in two representative patients (A+B)**. Although additional mutations developed in the progress of therapy (bold characters) the resistance factor for ATV and SQV decreased below the cut-off for full susceptibility in both patients compared to analyses one year before. Antiretroviral drugs are illustrated with corresponding resistance factors (cut-off: 0-3.5 = sensitive 3.6-9.5 (29 for LPV) = intermediate; >9.0 (29 for LPV) = resistant). Genotypic resistance interpretations derived from five common online tools showed considerable discrepancies in weighting of ATV and SQV resistance levels compared to each other and to phenotypic results (grey and white colour). **A) **One patient with failing APV containing therapy after week 72. **B) **Another patient with a failing IDV/LPV treatment before start of SQV containing therapy.

At first, the impact of L76V on ATV- and SQV-resistance characteristics was assessed before and after establishment of the mutation. Due to the manifestation of the L76V mutation as well as other minor mutations at resistance-relevant sites in the course of treatment, genotype-based interpretation tools predicted intermediate or mostly complete resistance against all PIs including ATV and SQV and the majority of NRTIs and NNRTIs resulting in an active drug score (ADS) of ≤ 1.0 for the failing regimen (Figure 1). Interestingly, in phenotypic analysis, the resistance factor (RF) for ATV and SQV remained at full susceptibility in both patients and even decreased for SQV from 31 to 1.1 (Figure 1A) and 1.1 to 0.6 (Figure 1B) and for ATV from 62 to 2.8 and 4.3 to 0.9, respectively.

**Figure 2:**
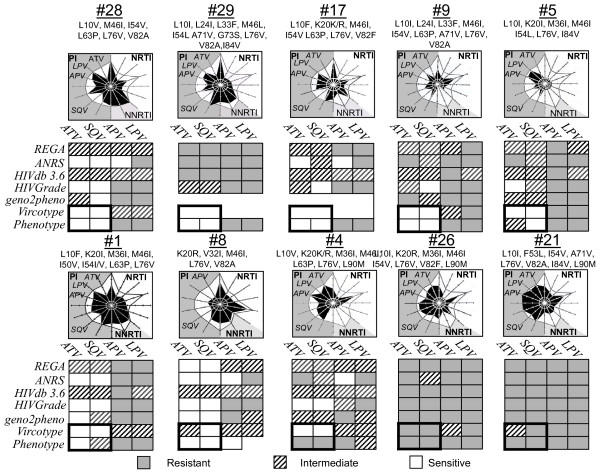
**The "resensitizing" effect of 76V could be observed in a variety of other patients before start of PI-containing therapy**. Genotypic and phenotypic data of 10 representative PI-experienced patients were analysed by using five common resistance interpretation systems Stanford HIVdb 4.3.6; REGA v7.1.1, HIV-Grade 04/2008, ANRS 10/2007 and geno2pheno. Genotypic resistance results were compared to phenotypic resistance results derived from recombinant virus assay results and/or Virtual Phenotype™ analysis (Virco).

In a further aspect, genotypic and phenotypic resistance data of 10 patients, all L76V positive, was assessed in order to analyze if these observed resensitizing effects represent ubiquitous drug resistance patterns. Figure 2 supports this hypothesis on a variety of other patients harbouring HIV populations with L76V mutation. The accuracy and concordance of predicted genotype-based interpretations were compared with obtained phenotypic resistance levels from recombinant virus assays and virtual phenotype analysis.

Despite a general concordance in genotype- and phenotype-based resistance predictions for LPV and APV, there were wide discrepancies in the weighting of resistance for ATV and SQV, mostly overestimation of resistance in genotype-based predictions (Figure 2). However, most phenotypic resistance interpretations uncovered full susceptibility for the drugs ATV and SQV. In most cases L76V appeared to be associated with a variety of other resistance relevant protease mutations without effecting the resensitizing effect. However, in particular, the copresence of the protease mutation L90 M was notably associated with high ATV and SQV resistance factors (Figure 2; #4, #26, #21).

### Clinical outcome and follow-up in patients with L76V-adapted therapy

Considering the effect of L76V on susceptibility for ATV and SQV, the big question was obviously, how this mutation might affect the therapeutic option and strategy for patients with a narrow margin of remaining active drugs. A considerable issue remained to generalize data from a small cohort of patients with diverse optimized backbone therapies. Thus, this work focused on the amount of active drugs in the treatment of each patient. Table 1 shows the L76V-adjusted follow-up therapies that were administered after resistance prediction results.

**Table 1:** Active drug score (ADS) for the follow-up therapy

	**GROUP A**										**GROUP B**																	
	
**Pat-ID**	**#1**	**#2**	**#4**	**#12**	**#14**	**#17**	**#18**	**#21**	**#22**	**#46**	**#3**	**#5**	**#8**	**#9**	**#10**	**#11**	**#16**	**#19**	**#24**	**#25**	**#26**	**#27**	**#31**	**#33**	**#37**	**#39**	**#40**	**#41**
	
***Therapy***	*ATV SQV RTV FTC*	*ATV SQV* *RTV 3TC*	*ATV SQV RTV*	*ATV/r SQV* *3TC* *T20*	*SQV* *TPV/r* *3TC* *ddI*	*ddI* *d4T* *SQV*	*ATV* *SQV* *3TC* *T20*	*ATV* *EFV* *TDF* *AZT* *3TC* *MVC*	*ATV/r* *SQV* *AZT* *3TC* *TDF*	SQV/r TDF FTC	*LPV/r* *SQV* *3TC* *d4T* *TDF*	*LPV/r* *ATV* *AZT* *3TC*	*LPV/r* *SQV* *d4T*	*LPV/r* *SQV* *ENF*	*LPV* *ATV* *FTC* *TDF*	*LPV/r* *ATV* *EFV*	LPV/r SQV	*LPV/r* *SQV*	APV SQV TDF	APV SQV ddC d4T	*APV/r* *SQV*	*LPV/r* *SQV* *FTC* *TDF*	*LPV/r* *SQV* *3TC* *ETR*	*LPV/r* *SQV* *AZT* *3TC* *TDF*	*LPV/r* *SQV* *ddI* *3TC*	*LPV/r* *SQV* *TDF*	*LPV/r* *SQV* *TDF* *MVC*	*LPV/r* *ATV* *TDF* *AZT* *ABC* *3TC*
	
***Rev.-Transcriptase mutations***	41L 44D 67N 98G 103N 118I 210F 215Y 219R	41L 67ss 69S 188L 215Y	41L 118I 184V 215Y	41L 44A 67N 75I 103N 108I 118I 210W 215Y	41L 67N 69D 70R 74V 103N 181C 210W 215F 219Q	67N 70R 103NS 190A 184V 219Q	41L 67N 69E 75I 103N 108I 118I 178M 210W 215Y	41L 67N 74V 101Q 184V 215Y	41L 44D 67N 101E 103N 118I 184V 210W 215Y 219E	n.d.	41L 67ss 69S 101Q 181C 190S 215Y	65R 70R 103N 108I 115F 151M 179E 184V 219E	67N 184V 210W 215Y 219Q	41L 44D 67N 75L 118I 181C 184V 190A 210S 215Y	41L 44D 67N 70R 190A 227L 184V 210W 215F 219R	41L 67N 74V 98G 118I 184V 210W 215Y 227L	41L 44D 103N 118I 184V 210W 215Y	no data	no data	no data	67N 70R 103S 184V 190A 215F 219Q	41L 67N 75I 118I 210W 215F	41L 44A 67N 75M 101Q 118I 184V 210W 215F	41L 74V 101Q 103N 108I 181C 190A 210W 215Y	41L 44D 67G 103N 118I 184V 210W 215F	67N 70R 215I 219Q	67N 70R 215I 219Q	41L 67N 70R 184V
***Protease mutations***	10F 20I 36I 46I 50V 54I/V 76V	10V 46I 47V 71V 76V 77I	10V 20R 36I 46I 76V 90M	10I 33F 46L 76V 82F 90M	10V 20R 33F 36I 54V 73S 76V 90M	10F 20R 46I 54V 63P 76V 82F	10I 33F 36L 46L 76V 82F 84V 90M	10I 53L 54V 71V 76V 77I 82A 84V 90M	10I 33F 46L 54V 71I 76V 77L 82A 90M	10I 33V 60E 76V	10V 46L 54V 63P 71V 82A 93L	10I 20I 36I 46I 54L 76V 84V	20R 32I 46I 76V 82A	10I 24I 33F 46I 54V 63P 71V 76V 82A	10V 20I 36I 46I 47V 53L 76V 84V 90M	10F 33F 46L 54L 71V 76V 77I 84V 90M	10F 46I 54M 71V 76V 82A	10I 20R 24I 36I 46I 54V 76V 82C	10F 33F 54V 71V 76V 77I 82A	10I 20R 35D 46I 54V 76V	10I 20R 36I 46I 54V 71V 76V 82F 90M	10I 46I 47V 71V 76V 90M	10I 13V 32I 33F 36I 46I 76V 84V 90M	10F 46I 47V 76V 84V	10R 32I 33F 46I 47V 76V 84V 88S 90M	10F 20I 36I 46I 76V 84V	10V 13V 24I 33F 46I 54V 76V 82A	20I 36I 54V 76V 82A
	
**Active Drug Scores**																												
*HIVdb 4.3.6*	**[1.5]**	(2.25)	0.5	**[1.75]**	0.75	1,25	**[1.5]**	**[1.75]**	0.25	3.0	1,5	**[0.5]**	**[0.75]**	1.25	0.25	**[0.75]**	**[0.5]**	**[0.5]**	1.5	n.d.	0.5	**[1.25]**	**[0.25]**	**[1.5]**	0.0	**[1.0]**	**[1.75]**	**[1.75]**
*Rega V7.1.1*	2.0	(2.75)	1.5	2.0	1.0	1.0	2.0	3.5	0.0	3.0	1.75	**[1.0]**	**[1.5]**	1.0	0.0	**[0.25]**	**[0.75]**	**[0.75]**	1.75	n.d.	0.75	**[1.0]**	**[0.5]**	2.75	0.5	2.0	2.25	4.25
*HIVGrade04/08*	2.5	(3.0)	1.5	2.25	1.0	0.5	2.75	2.75	0.0	3.0	(2.0)	**[1.0]**	**[1.0]**	(2.0)	0.0	**[1.0]**	**[1.0]**	**[1.0]**	(2.0)	n.d.	1.0	**[1.75]**	**[0.0]**	[**1.75**]	0.0	**[0.75]**	2.0	2.0
*ANRS 10/2007*	3.0	(2.0)	0.5	**[1.0]**	1.0	1.0	2.0	2.5	0.5	3.0	1.5	**[0.0]**	2.0	1.0	0.5	**[1.0]**	**[1.0]**	**[0.5]**	(2.0)	n.d.	0.5	2.0	**[0.5]**	2.5	1.5	**[1.5]**	2.5	3.0
geno2pheno	2.0	(3.0)	1.0	2.5	(2.0)	n.d.	n.d.	2.0	n.d.	3.0	(2.0)	**[0.5]**	**[0.5]**	1.5	0.0	**[1.0]**	n.d.	n.d.	n.d.	n.d.	0.0	2.5	n.d.	2.0	0.0	1.0	2.0	4.0
VircoType	2.25	(2.0)	(2.0)	2.5	1.5	n.d.	n.d.	3.5	n.d.	3.0	2.5	2.0	2.0	1.0	0.5	**[0.5]**	**[1.0]**	**[1.0]**	n.d.	n.d.	0.5	**[1.0]**	**[1.0]**	3.0	1.5	2.0	3.0	3.5
***Phenotype***	2.5	n.d.	0.0	n.d.	n.d.	(3.0)	3.0	3.0*	n.d.	n.d.	n.d.	2.5	2.5	(2.0)	n.d.	n.d.	n.d.	**[0.5]**	n.d.	n.d.	0.0	n.d.	n.d.	n.d.	n.d.	n.d.	n.d.	n.d.
	
**Follow-up**																												
***Baseline VL***	1070	160000	26900	3231	47653	859000	7700	27000	35400	4248	6680	25900	4840	31400	39000	20163	8751	8800	88100	16100	310510	< 50	72100	14900	1300	744	2078	443
***Lowest VL (12-96 weeks)***	< 50	3410	38700	< 50	4943	190	< 50	< 50	1020	< 50	1320	< 50	< 50	116	24000	< 50	< 50	< 50	559	< 50	18270	< 50	< 50	< 50	1300	< 50	< 50	< 50
***VL at week 96***	13589	46570	152000	< 50	20626	LFU	LFU	LFU	16500	LFU	LFU	< 50	< 50	116	LFU	< 50	< 50	LFU	LFU	1777	LFU	391	60	< 50	LFU	< 50	< 50	< 50

Based on five genotypic resistance interpretation tools as well as two phenotypic resistance tests, an active drug score (ADS) for the follow-up therapy was calculated by adding the activity score for every active drug ranging from AS = 1.0 for complete sensitivity, AS = 0.75 for potential low-level resistance, AS = 0.5 for intermediate resistance, AS = 0.25 for possible resistance and AS = 0.0 for complete resistance. Their prediction on follow-up therapy was then compared with the virological response in a time frame of 96 weeks. Sucessfull therapies despite an active drug score prediction of <2 are displayed in bold and square brackets. Unsucessfull therapies despite an active drug score prediction of ≥2 are displayed in round brackets. LFU=loss of follow-up.

**Figure 3:**
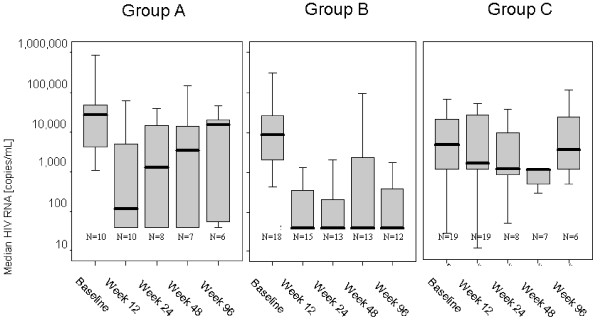
**Clinical outcome and suppression of viral load in a time frame of 96 weeks of follow-up therapy (illustrated as box plot figure)**. Median viral loads are illustrated in bold lines in between the upper- and lower quartile. Group B (ATV and/or SQV plus LPV or APV containing treatment) show a higher long-term success rate after 96 weeks of follow-up therapy in comparison to group A (ATV and or SQV without L76V-selecting drug).

Sufficient virus suppression below the detection limit was initially observed in 50% of group A (ATV and/or SQV without L76V-selecting drug) and 67% of group B (ATV and/or SQV plus L76V-selecting drug LPV or APV) within the first weeks of follow-up therapy. Despite similar response rates at first, a sustained therapy success with virus suppression still below 50 copies/mL at week 96 and longer was predominantly achieved in group B patients where the selection pressure on L76V was constantly maintained by the drugs LPV or APV (Table 1, lower rows). While 66.7% of group B patients remained under detection levels at week 96, there was a significantly lower success rate in group A patients with 16.7% remaining <50 copies/mL in per-protocol analysis (p = 0.002; χ^2 ^test) (Table 2 and Figure 3). Patients of group C did not show any virus suppression below detection limit.

**Table 2:** Comparison of results (group A and group B).

	Virus Suppression			Therapy success		
	
	Group A Median Viral load	Group B Median Viral load	Mann-Whitney	Group A Success	Group B Success	χ2
Baseline	26,950 (N = 10)	8,800 (N = 17)	P = 0.12	0% (N = 10)	5.9% (N = 17)	P = 0.998

Week 12	370 (N = 10)	< 50 (N = 15)	P = 0.07	40.0% (N = 10)	66.7% (N = 15)	**P = 0.035**

Week 24	4650 (N = 8)	< 50 (N = 13)	P = 0.16	37.5% (N = 8)	69.2% (N = 13)	P = 0.166

Week 48	3410 (N = 7)	< 50 (N = 13)	P = 0.19	42.9% (N = 7)	53.8% (N = 13)	P = 0.425

Week 96	15,045 (N = 6)	< 50 (N = 9)	**P = 0.044**	16.7% (N = 6)	54.5% (N = 9)	**P = 0.002**

Comparison of results (group A and group B). Median viral loads and the therapy success rates illustrate a significantly better long-term suppression of HIV when SQV/ATV plus optimized backbone therapy is combined with a L76V-selecting drug. In bivariate analysis, these results were independent from slightly different baseline viral loads ( < 0,5log) between group A and B.

Interestingly, despite a successful virus suppression <50 copies/mL, the majority of group B therapies were expected to have an ADS below 2.0 after genotypic resistance predictions, indicating a very likely event of therapy failure (bold numbers in square brackets) (Table 1).

Most of the cases where therapies were predicted to have active drug scores of ≥2.0 turned out to be successful. Only few experienced virological failure despite an ADS of more than 2.0 (indicated in round brackets).

A major question remained obviously, why patients of group A display earlier therapy failures than patients of group B. After two years of follow-up therapy, only one patient of group A, who additionally received a fusion-inhibitor containing treatment, showed a viral load still below 50 copies/mL. The median viral load increased after 24 weeks of follow-up in group A (Figure 3). More interestingly, due to a loss of selection pressure on the L76V mutation, it was then undetectable in those patients who failed therapy, resulting in a decrease of the ADS below <2.0 (Table 3). While L76V was undetectable in patients where no L76V-selecting drug was applied, it persisted in group B and group C where selection pressure on mutation L76V was maintained (Table 3). In these patients the therapy failure had other reasons (e.g. acquisition of L90M).

**Table 3:** Compensatory changes in virus genotypes within 96 weeks of follow-up therapy.

Group	Patient ID	Protease mutations at start of therapy	Time of therapy failure	Time of 2nd genotype	Protease mutations after therapy failure
**A**	#1	L10FL, K20I, M36I, M46I, I50V, I54IV, L63P, **L76V**	Week 48	Week 144	L10F, V11I, I13V, K20R, V32I, L33F, M36I, M46I, I47V, I54M, L63P, A71V, G73S, I84V, L90M
	#2	L10V, M46I, I47V, L63P, A71V, **L76V**, V77I	Week 12	Week 48	L63P, V77I (therapy interruption)
	#4	L10V, K20RK, M36I, M46I, L63P, L76V, L90M	Week 12	Week 24	L10V, K20R, M36I, M46I, F53L, L63P, I84IV, L90M
	#22	L10I, L33F, M46L, I54V, L63P, A71I, **L76V**, V77I, V82A, L90M	Week 12	Week 48	L10I, L33F, M46L, F53L, I54V, L63P, A71T, G73S, V77I, V82A, L90M

**B**	#8	K20R, V32I, M46I, **L76V**, V82A	Week 48	Week 48	K20R, V32I, M36I, M46I, F53FL, **L76V**, V82A, L90LM
	#9	L10I, L24I, L33F, M46I, I54V, L63P, A71V, **L76V**, V82A	Week 12	Week 24	L10I, L24I, L33F, M46I, F53L, I54V, L63P, A71V, **L76V**, V82A, I84V
	#10	L10IV, K20I, M36I, M46I, I47V, F53L, L63P, A71V, G73 D, **L76V**, I84V, L90M	Week 12 compliance	Week 48	L10V, K20I, L33I, M36I, M46I, I47V, F53L, L63P, A71V, G73 D, **L76V**, I84V, L90M
	#27	L10I, M46I, I47V, L63P, A71V, **L76V**, L90M	Week 48	Week 48	L10I, M46I, I47V, L63P, A71V, **L76V**, I84V, L90M

**C**	#6	L10V, L33F, M46L, I54V, A71V, L63P, A71V, **L76V**, V82A	Week 12	Week 96	L10V, K20R, L33F, M36I, M46L, I54V, A71V, **L76V**, V82A
	#23	L10FIRV, L33F, I54MV, D60E, L63P, A71V, **L76V**, V82F	Week 12	Week 24	L10FIRV, L33F, I54MV, D60E, L63P, A71T/V, **L76V**, V82F

Compensatory changes in virus genotypes within 96 weeks of follow-up therapy. Patients with failing therapies within the 96 weeks received a second resistance testing. While L76V was still present in patients receiving L76V-selecting drugs, it was then absent in patients without these drugs (new detected mutation are underlined). Therapy failure in group B was noticeable associated with an additional establishment of the protease mutation L90 M.

These results additionally indicate benefits for patients with L76V-selecting drugs in combination with L76V-"resensitized" drugs. A major issue remains the establishment of additional protease gene mutations i.e. L90 M and further compensatory changes over the time (Figure 2; #4, #21, #26), making it crucial to suppress the virus completely and monitor viral load in close intervals.

## Discussion

Little is known about the impact of drug-resensitizing mutations on antiretroviral therapy. Most works mainly describe the effects of resistance mutations on reductions in drug susceptibility. However, selective pressure of drug therapy may also lead to shifts in the quasispecies distribution and fitness of those mutants with decreased sensitivity to the respective antiretrovirals [11,12]. This loss in replication fitness may be even larger for a multi-drug resistant virus and might lead to a better starting point for particular antiretroviral regimens [13]. Nevertheless, it is not always applicable that the acquisition of drug-resistance mutations inevitably result in loss of viral fitness. Even in case a loss is apparent, the virus may select compensatory changes over time [12,14]. This may explain why current treatment guidelines still advocate a switch to antiretroviral treatment regimens following the emergence of drug resistance mutations and possibly prior to selection of compensatory changes [11]. In summary, drug hypersusceptibility mutations which reduce viral fitness are difficult to maintain in the predominant virus population in multiple pretreated patient.

In this article we provide insights into a possibility how to maintain efficient selection pressure on the protease mutation at position L76V by combining one drug, which selects L76V (in this case LPV, APV, DRV) and another drug which gains efficiency when L76V develops. This article reports about a significant clinical benefit of the protease mutation L76V on drug susceptibility to ATV and SQV due to resensitizing effects in multi-resistant patients resulting in a significantly higher long-term therapy success. These results may be in line with explanations from molecular dynamics- and free energy studies recently reported by Alcaro et al. who found that in the presence of the L76V substitution, ATV reveals a more productive binding affinity, in agreement with hypersusceptibily data [15].

## Conclusion

The strategy of combining mutation-selecting drugs with "resensitized" drugs has already been discussed for the reverse transcriptase mutation M184V in NRTI-containing therapies [6,13,16] and has also been shown to be an adequate option for a couple of other mutations including the mutation N88 S [10].

Despite initially adequate therapy response rates in 50% (group A) - 67.7% (group B) of cases, it remains a major issue that virological failure under these therapies often occur due to compensatory changes in the virus genotype over the time mostly due to an additional establishment of further mutations in the respective gene [12,14]. As shown in Table 3, failure of therapy in L76V-positive patients with ATV and/or SQV containing therapy was noticeable associated with an additional establishment of the protease gene mutation at position L90 M which resulted in resistance against all available PIs [17]. Six of eight patients who received a second genotypic resistance test following therapy failure were diagnosed positive for L90 M. The remaining two were therapy non-compliant. Thus, it might be questionable if SQV, which primarily selects L90 M should be replaced in favour of ATV [18-20]. In addition, due to the approval of new drug classes over the past years one might err on the side of caution to supplement therapy regimens with new drugs. Due to the low potency of the present cohort and varying amounts of HIV non-B infected individuals in the examined patient groups, caution should be additionally advised, since these limitations might have effect on clinical outcomes.

Nevertheless, since there are still distinct discrepancies, mostly overestimation of resistance, in the prediction of the resistance level for Atazanavir and Saquinavir in five of the most common genotypic interpretation systems, there is still a need for further evaluation in the case of L76V occurrence.

## Methods

### Clinical material

**Figure 4:**
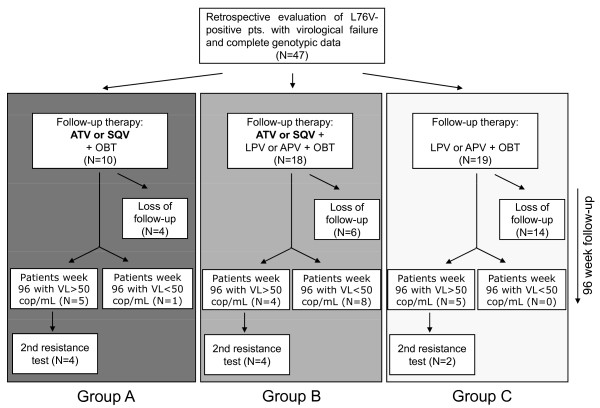
**Inclusion criterion for the retrospective analysis of 47 L76V-positive patients**.

HIV strains of 46 intensely pretreated (34 showed NRTI/NNRTI/PI resistance/12 showed NRTI/PI resistance) and one naïve with transmitted mutation, L76V-positive patients derived from 24 centres throughout Germany between 1999-2009 were retrospectively analysed for HIV-resistance patterns and success of follow-up therapy. The inclusion criterion is provided in Figure 4. Descriptional statistics concerning person-to-person variations of virological and immunological parameters were assessed at baseline before switch of therapy and are provided in Table 4.

**Table 4:** Patient characteristics and parameters.

Parameter	Total	Group A (N = 10)	Group B (N = 18)	Group C (N = 19)
**Gender**				
Male	84%	100%	100%	68,7%
Female	16%	0%	0%	31,3%
**HIV-1 subtype**				
Patients with subtype B	69%	80%	64%	67%
Patients with non-B subtype	31%	20%	36%	33%
**Treatment history**				
Mean duration under ART in months (mean)	66	80	88	54
Current active drug score (mean)	-	1.5	1.3	0.5
**HIV-1 RNA [copies/ml; median]**				
Baseline	20,163	26,950	8,800	42,600
**CD4 cell counts [cells/μl; median]**				
Baseline	260	291	307	246

Patient characteristics and parameters. All patients were categorized in three groups as described throughout the article: Group A (ATV and or SQV), group B (ATV and or SQV plus L76V selecting drug LPV, APV or DRV) and group C (L76V selecting drug plus optimized backbone therapy).

All patient data was categorized in three groups concerning the follow-up therapy:

Group A: ATV and/or SQV (no selection pressure on L76V)

Group B: LPV or APV plus ATV or SQV (maintained selection pressure on L76V)

Group C: LPV or APV plus other drugs (maintained selection pressure on L76V)

All patients received an optimized backbone therapy.

### HIV-1 RNA Quantification

Plasma of patients was analysed at baseline and week 12, 24, 48 until end of investigation at week 96 to monitor efficiency of therapy. Plasma RNA was measured by using the COBAS AMPLICOR HIV-1 Monitor system and the Abbott m2000sp/rt system according to the manufacturer's recommendations.

### Genotypic resistance testing

Plasma samples of all 47 patients were collected and stored at -20°C until time of RNA extraction. All specimens were processed by using the FDA-approved Siemens HIV TruGene system as well as the Abbott HIV-1 Genotyping System on the Applied Biosystems' 3100 capillary electrophoresis platform according to the manufacturer's recommendations. HIV-1 genotypes were processed and analyzed by using the wildtype LAV-1 sequence as reference. The sensitivity for detecting minor quasispecies variants was 15%. In this cohort, only patients with major L76V positive population were included. Minor wildtype variants were not detected at this position.

### Phenotypic resistance testing

Phenotypic resistance analysis of the complete protease gene and the first 900bp of the RT were performed according to an earlier described recombinant virus assay by determining a virus specific resistance factor [21]. In addition, the Virtual Phenotype™(based on 53,000 paired genotypes and phenotypes) from Virco was assessed for those patient samples where no recombinant virus assay was realizable.

### Interpretation of drug resistance

Several algorithms are available worldwide, both in public and private domains. The concordance of resistance predictions was analysed between the five most commonly used algorithms [REGA v7.1.1 [22] HIVGrade ver.12/2008 [23] ANRS ver.10/2007 [24] Stanford HIVdb ver.4.3.6 [25] and the geno2pheno online tool [26]] for the drugs ATV, SQV, LPV and/or APV. Multiple resistance tests in treatment history were cumulatively documented. In addition, an active drug score (ADS) was determined in order to analyze the amount of remaining active drugs in follow-up therapies of each patient (susceptible = +1/low intermediate = +0.75/intermediate = +0.5/high intermediate = +0.25/resistant = +0.0). This ADS allowed a statement concerning the prediction of follow-up therapy. It is generally accepted that a successful therapy should contain at least two active drugs, preferably three (ADS ≥2.0) [27,28].

## Competing interests

The authors declare that they have no competing interests.

## Authors' contributions

FW has made substantive intellectual contribution to the study including acquisition-, analysis- and interpretation of data and finally drafting the manuscript. JV assisted as consultant in patient-specific aspects and was involved in manuscript revision. GN was responsible with genotyping processes as described in the manuscript. RE was responsible for genotypic resistance interpretation and manuscript revision. HW realized the phenotypic resistance analysis. PB and AT assissted in concept and design aspects and directed sample- and data acquisition. HK, RK, MS and TB were significantly involved in data acquisition, provision of samples and manuscript revision. All authors read and approved the final manuscript.
